# Complete Genome Sequence of *Cellulomonas* sp. Strain C5510, Isolated from Pacific Ocean Seawater

**DOI:** 10.1128/mra.00857-21

**Published:** 2022-01-20

**Authors:** Jing Wang, Ting Chen, Ying Xu, Jiafan Jin, Tiantian Yu, Na Ren, Xiunuan Chen, Jifang Yang, Sidong Zhu, Jigang Chen

**Affiliations:** a College of Biological and Environmental Sciences, Zhejiang Wanli University, Ningbo, China; University of Southern California

## Abstract

We report the complete genome sequence of *Cellulomonas* sp. strain C5510, obtained by Oxford Nanopore and Illumina sequencing. The approximately 4.15-Mb genome sequence consists of one circular chromosome and one circular plasmid, with an overall G+C content of 74.9%.

## ANNOUNCEMENT

Strain C5510 was isolated from a seawater sample collected at a depth of 25 m in the Pacific Ocean (148.5°E, 9.3°N) using the dilution plating technique on International Streptomyces Project (ISP) medium number 5 (ISP5) agar (1) and maintained as glycerol stocks at −80°C.

The 16S rRNA gene of strain C5510 was first amplified by PCR using the primers 27F and 1492R (2) and then ligated into a pMD-18T vector via TA cloning for subsequent sequencing. Comparative analysis of the 16S rRNA gene sequence revealed that strain C5510 is closely related to members of the genus *Cellulomonas*, sharing 95.4 to 99.2% sequence similarity with the type strains of species of the genus *Cellulomonas* and most closely related to Cellulomonas hominis JCM 12133 (99.2% identity; GenBank accession no. BBHJ01000002).

For genomic DNA (gDNA) isolation, a single colony of C5510 was inoculated into Erlenmeyer flasks containing marine broth 2216 (BD Difco) and shaken aerobically at 30°C for 3 days. Bacterial cultures were collected, and a Wizard genomic DNA purification kit (Promega, Madison, WI, USA) was used for gDNA extraction for Illumina and Oxford Nanopore sequencing, following the manufacturer’s protocol. The purified gDNA was quantified using a TBS-380 fluorometer (Turner BioSystems Inc., Sunnyvale, CA). The DNA was used for Illumina and Oxford Nanopore sequencing. For Illumina sequencing, 1 μg gDNA was used with the NEXTflex Rapid DNA-Seq kit (Bioo Scientific, USA) according to the manufacturer’s instructions. The Illumina library was sequenced on the Illumina HiSeq X Ten platform at Majorbio (Shanghai, China). Approximately 1.6 Gb of raw data (150-bp paired-end reads) was generated. A total of 5,901,360 reads were subjected to control and trimming using SOAPnuke v1.3.0 (3), which removed reads containing 50% low-quality bases (quality value, ≤5) and overlaps with adapter sequences, generating a total of 5,776,098 clean reads.

For Nanopore sequencing, high-molecular-weight DNA was isolated using a BluePippin system (Sage Science, Beverly, MA, USA). Approximately 2.5 μg genomic DNA was used for library construction using a one-dimensional (1D) ligation sequencing kit (SQK-LSK109; Oxford Nanopore Technologies, Oxford, UK). No size selection or shearing was applied. The library was loaded into an R9.4 flow cell for the PromethION platform (Oxford Nanopore Technologies). After base calling using Guppy v4.0.15 (Oxford Nanopore Technologies), a total of 84,989 reads were obtained, with an average length of 16,282.5 bp and an *N*_50_ value of 27,500 bp. Nanopore quality control was achieved using NanoPlot v1.15.0 with a threshold value (Q) of >7 (4).

*De novo* hybrid assembly was performed with quality-checked Illumina and Nanopore reads using Unicycler v0.4.8 (5), yielding a 4,040,327-bp circular chromosome and a 110,066-bp circular plasmid with an overall G+C content of 74.9%. The final coverage of the genome was 100%. The depth of Illumina sequence averaged 423.9×, while for Nanopore, it averaged 389.0×. For all software, default parameters were used, except where otherwise noted. The genomic sequences were uploaded to NCBI and automatically annotated using the NCBI Prokaryotic Genome Annotation Pipeline v5.2 (6), which predicted a total of 3,802 gene sequences, including 3,710 coding sequences, 6 rRNAs, 45 tRNAs, 3 noncoding RNAs (ncRNAs), and 38 pseudogenes. Analysis using antiSMASH v6.0 (7) revealed that the C5110 genome contains five gene clusters involved in secondary metabolite production, including two polyketide synthase (PKS) enzymes with three canonical types, one lanthipeptide, one linaridin, and one terpene gene cluster.

### Data availability.

This whole-genome sequence has been deposited in NCBI under BioProject and BioSample accession no. PRJNA756105 and SAMN20855178, respectively, and GenBank accession no. CP081862.1 (chromosome) and CP081863.1 (plasmid). The SRA accession numbers of the Illumina and Nanopore reads are SRR15648130 and SRR15648129, respectively.
